# The microbiome of the nasopharynx

**DOI:** 10.1099/jmm.0.001368

**Published:** 2021-06-24

**Authors:** Matthew Flynn, James Dooley

**Affiliations:** ^1^​ School of Biomedical Sciences, Ulster University, Coleraine BT52 1SA, UK; ^2^​ Otolaryngology Department, Queen Elizabeth University Hospital, Glasgow G51 4TF, UK

**Keywords:** nasopharyngeal, microbiome, respiratory virus, bacterial infection

## Abstract

The nasopharyngeal microbiome is a dynamic microbial interface of the aerodigestive tract, and a diagnostic window in the fight against respiratory infections and antimicrobial resistance. As its constituent bacteria, viruses and mycobacteria become better understood and sampling accuracy improves, diagnostics of the nasopharynx could guide more personalized care of infections of surrounding areas including the lungs, ears and sinuses. This review will summarize the current literature from a clinical perspective and highlight its growing importance in diagnostics and infectious disease management.

## Introduction

As a microbiological niche, the nasopharynx (NP) demands an increased understanding of its dynamics, as the last ecological reservoir bordering the relatively microbially scarce lower respiratory tract, sinuses and middle ear [1–4]. Infections of these three sites respectively represent the leading cause of childhood and neonatal mortality worldwide [5], the second most commonly antimicrobial-overprescribed [6], and the most common reason to seek medical attention for under-5s in the USA [7]. In an era of antimicrobial resistance, translation of research from emerging molecular techniques and clinical stewardship measures will determine efficacious treatment [8, 9]. Whilst the oral flora are as logical a source of the microaspirations that seed lower airway disease as the NP, it seems to develop on a different taxonomic axis early in life [10–13]. Indeed, loss of oral/NP dissimilarity caused by, or associated with influx of oropharyngeal taxa into the NP precede respiratory tract infections (RTIs) [14]. As microbiological knowledge develops from a Kochian dichotomy of infection and health, the patient may in future be increasingly stratified within severity scales of disease or specific microbial profiles predictive of worse outcomes. The ‘Feverpain’ score stratifying patients into groups of relative risk of oropharyngeal streptococcal isolation based on symptomatology, and a classification model predicting Paediatric Intensive Care admission based on patient characteristics and isolated pathogens, are early examples of this [15, 16]. This review will describe the evolution of the healthy NP microbiome, its relevance to disease of the lower airways, sinuses and middle ear, and propose further areas of investigation and evidence synthesis.

### Colonisation over lifespan

In the first year of life the genera *Moraxella, Streptococcus, Corynebacterium, Staphylococcus, Haemophilus* and *Alloiococcus*/*Dolosigranulum* predominate, with likely ancestry from maternal skin, vaginal and breast milk progenitors [17, 18]. The NP rapidly develops as a distinct niche from the oral cavity with a seemingly protective increase in diversity [19]. Serial sampling of the NP in relatively healthy (>3 RTIs per year) children >1 yr with a showed early overgrowth with streptococcal spp., supplemented by *Corynebacterium* and *Dolosigranulum* with later colonization with *Moraxella* after 2–3 months. These roles were reversed in those with increased RTI frequency where *Moraxella* dominated earlier and *Corynebacterium* and *Dolosigranulum* remained less established [20]. Similarly, pre-term NPs are associated with within-group heterogeneity compared to the full term, a potential instability mimicking that seen in ensuing Rhinovirus infection, notably decreased abundance of *Corynebacterium* and *Alloiococcus* [21]. At 18 months, *Enhydrobacter* replaces streptococcal spp. when describing the six predominating operational taxonomic units (OTUs) (defined as being present in over 50% of nasopharyngeal samples). Indeed *Proteobacteria*, along with *Fusobacteria* and *Cyanobacteria*, achieve a seasonal abundance in autumn/winter that is lost by spring, which invites transient growth of *Bacillus, Brevibacillus, Lactobacillus* and *Bacteroidetes*. In this study, the overall microbial diversity of the nasopharynx did not significantly fluctuate between autumn, winter and spring [22]. Over childhood and into adulthood there develops a richness in NP taxa, accompanied by increased evenness [23] and diversity in neighbouring oropharyngeal flora, relative to adults and elderly presenting to the emergency department with pneumonia [24]. Within adults aged 50–80, there is a greater absolute number of pathogens extracted by swabs in men compared to women [25]. The topographical dissimilarity between the anterior nares and oropharynx seen in the middle aged is lost within the elderly population; such transitions in microbiome may precipitate or avail of increased susceptibility to disease in this population [26]. This mimics the loss of variance between the oral and nasopharyngeal diversity associated with predisposition to disease early in life [27]. However larger more longitudinal studies within the adult population are required to describe the vectors therein.

### Environmental/aetiological factors influencing NP microbiome composition

The evolution of the healthy microbiome cannot be reduced to a gradual collection of key OTUs upon the epithelial seabed. Many other aetiological and iatrogenic factors affect its development. Breastfeeding showed a significant change in the 6 week microbiome compared with formula feeding, notably with increased presence and abundance of classically commensal *Dolosigranulum* and *Corynebacterium* [28]. Significant decrease in abundance of these two potentially keystone species is noticed in infants who had antibiotic use in the preceding 4 weeks before sampling [29]. Short-term corticosteroid inhalation was not found to significantly alter the nasopharyngeal microbiome, but longer-term studies are needed [30]. Smoking appears to have a positive impact on the raw incidence of known pathogenic genera, and suppressing key ‘interfering’ species, but many of these studies have been underpowered [31, 32]. Lower socioeconomic indicators correlate with increased prevalence of *M. catarrhalis, S. aureus* and antibiotic-resistant *S. pneumoniae*, and epidemiological factors such as older siblings, daycare attendance and rural occupancy exerted a positive pressure toward pathogen carriage [33, 34]. These studies were conducted with traditional culture, not quantitative and sensitive molecular techniques however, therefore potential for false negatives is high. Pig farming has a positive and unsurprising effect on nasopharyngeal diversity indicating that the external environment has a key role in determining the final NP microbiome composition [35]. In line with similar findings in the gingivae and dental plaques, bacterial biofilms have been established as a mode of survival on the adenoidal surface [36–38]. As a sustaining and protective extracellular resin, biofilm is emerging as a necessary *in vivo* concept with relevance to therapy and microbial synergy in understanding the microbiome in health [39]. Whether or not biofilms are a driver of respiratory disease is yet to be established [40]. This rise in incidence of pathobionts is also noticed with lower lean body mass to fat ratios in men and higher waist-to-hip ratios in females, both considered markers of fertility and immune competence [41]. Immunomodulatory effects are noticed in raised serum Vitamin D levels, which reduce self-reported symptoms of the upper RTI: ears, sinuses, malaise and use of antibiotics in the immunosuppressed, and Vitamin D also augments dendrocyte maturation and matriculation against pneumococcal peptidoglycan *in vitro* [42, 43]. The asthmatic core microbiome identified in a study with mean age 11 years mimicked the previously described core microbiome at 18 months, but without *Enhydrobacter* and with *Moraxella, Haemophilus* and *Streptococcus* being observed in 95% of samples, the same trio previously noticed in Rhinovirus susceptible infants [44, 45]. There may be further unknown pharmacological, meteorological or behavioral pressures as yet unstudied.

### Pathogens, pathobionts and commensals: changing roles for the NP microbiome

The diverse microbial landscape is further involved by the relative pathogenicity of organisms, with microbes traversing the commensal-pathogen continuum depending on circumstance and coinfection. Pneumococcus for instance, commonly considered the main causative agent of pneumonia, may be considered a member of the healthy nasopharynx [46], and conversely species considered normal commensals may be implicated in severe disease of the immunocompromised [47]. The introduction of the pneumococcal vaccine has reduced disease burden, but has led to serotype replacement of *S. pneumonia*, and immediate epidemiological shifts in carriage of non-typable *Haemophilus influenzae* [48, 49]. Similarly *Moraxella* spp., long considered a benign human symbiont, has been implicated in a consistent percentage of middle ear and sinus infections, and are an important cause of exacerbations of chronic obstructive pulmonary disease [50]. Colonization rates, determined by culture, of *S. pneumoniae*, *S. aureus* and *M. catarrhalis* were significantly higher in patients with variant types of mannose-binding lectin, Toll-like receptor 2 (TLR2) and TLR4, respectively, receptors upregulating the innate immune system derived from polymorphic alleles, suggesting a genetic basis for variable colonization [51]. The underlying bacteria–bacteria and virus–bacteria and immune–bacterial interactions are complex, and the exact link between competition, overgrowth and disease manifestation requires considerable future study [52, 53].

### The virome and mycobacteriome in health

The NP virome is a common cause of upper respiratory illness. Metagenomic analysis of NP swabs yielded a mean 86 viral sequences per sample in children under 3 with unexplained fever, compared with 56 from health controls, as well as greater richness and diversity [54]. This greater yield was a contradiction to previous purely PCR-based studies with little or no viral load detected in normal controls, controls which though significantly predict health, suggest a benign carriage akin to the commensal bacteriome [55, 56]. This high sensitivity of viral presence in under 3s via next-generation sequencing was replicated in a second case-controlled study, where 71.2 % ‘healthy’ NPs contained viral nucleic acid compared with 94.4% of children with recurrent otitis media, with Polyomavirus, Bocavirus and Rhinovirus prevailing in health, the latter with incidence as high as 42.4% [57]. The Anelloviridae family however has been identified as the most prevalent in febrile children on metagenomic analysis [58]. Anthropologically, the presence of various Rhinovirus strains occur between aboriginal and non-aboriginal children at different rates, and are associated with *Moraxella* and *H. influenzae* within both populations, pathogens deemed responsible for otitis media [59, 60]. *Haemophilus* is further overrepresented in infants hospitalized with Respiratory Syncytial Virus (RSV) and drives response of mucosal cytokine CXCL8, while clearance of RSV is delayed in infants with a *Haemophilus* dominated NP microbiome [61, 62]. More recently, comparison of COVID-19 specimen collections under strict conditions favoured the NP as more likely to yield the virus than the oropharynx [63]. NP dysbiosis has also been associated with disease severity in *Mycoplasma pneumoniae* pneumonia compared with healthy controls [64]. Fungal disease is not normally implicated in the NP being unique to the paranasal sinuses [65].

### Lung, sinus and gastrointestinal relationships to the NP microbiome

The microbiome of the lung has been described as ecologically similar to the NP. The relatively abundant phylae *Bacteroides, Firmicutes* and *Proteobacteria*, however, have been a subject of doubt, whether selectively repatriated from the upper respiratory tract, or selectively contaminated whilst sampling the oropharynx [66]. Sterile dissection of healthy smokers' lungs have confirmed this microbiome, distinct from the oral cavity or nasopharynx, and more robust models of sterile sampling and controls for lavage contamination and oropharyngeal sampling confirm specifically lung-enriched organisms [67, 68]. As well as a source of emissary pathogens to the lungs, the NP microbiome may offer a diagnostic window to the rest of the respiratory tract. Furthermore, prevalence of keystone species at this level may gate downstream transmission of pathogens by colonization resistance [69]. Asthma control tests, an indication of how well asthma has been controlled over the preceding 4 weeks, was significantly lower in subjects with no viruses detected on NP swabbing than those with detected viruses [70]. NP swabs clear of viruses demonstrated lower NP microbiota compositions vary not only with RTIs but gastrointestinal infections also, inferring synergy of the wider metabiome [71]. Acute rhinosinusitis (RSS) is marked by symptoms of nasal congestion and nasal discharge, or facial pain or anosmia [72]. Prior history of RSS was associated with a significant depletion of NP taxa from over 100 genera, with only the species *Moraxella nonliquefaciens* demonstrating an increase in relative abundance [73]. Longitudinal studies through acute disease are required to describe dynamics more fully, but such statistically outlying OTUs, if detected on a consistent basis, may serve as biomarkers to predict or confirm disease. The NP acting as a reservoir for sinus infection model has been supported by a conventional culture study linking successfully detected pathogens at the osteomeatal complex in the mid-nasal cavity to a >90% coincidence in the NP [74]. More recent gene sequencing of microbiota from functional endoscopic sinus surgery patients found a microbiome similar to the anterior nares (AN), which became transiently similar to the NP at the time of operation, and then regressed to its original niche equilibrium after 6 weeks [75]. Dissimilarity between the almost isolated sphenoid sinus and the rest of the nose, manifests as increased detection rates of resistant commensals and anaerobes, however this study was underpowered and used culture techniques [76]. Multidisciplinary collaboration between clinicians and scientists along both respiratory and age continua will be required to further characterize their dynamics and provide clinical application.

### Otitis media

The otitis media (OM) culprit pathobiont triad in the conventional culture era were *Haemophilus influenzae, Moraxella catarrhalis* and *Streptococcus pneumoniae*, joined by *Alloiococcus otiditis* with PCR-based diagnostics, a pathogen so ubiquitous in the middle ear of previously antibiotic treated children as to raise suspicion of a facultative or saphrotrophic nature [77–79]. *Alloiococcus*, however, was found to be absent in the NP and predominating in cultures of middle ear fluid from perforated eardrums, suggesting its external ear origin [80–82]. The NP of children with OM, in whom it is relatively prevalent, is implicated with the above three classically cultured pathogens [83]. Quantitative-PCR detected the prevalence of *Haemophilus* in OM approaching the 90% NP detection rate of the same pathogen, a correlation not shared by *Moraxella* and *Streptococcus* spp. [84]. Next-generation sequencing of the NP in recurrent acute OM patients showed increased abundance of *Corynebacterium* and *Dolosigranulum* in healthy controls, with a lesser role played by *Moraxella. Dolosigranulum pigrum* is closely related to *Alloiococcus*, and *Corynebacterium* is from the same family as the otopathogen *Turicella*, suggesting possible colonization resistance for at least these genera [85]. These two, however, are very successfully inhibited by β-lactam antibiotics, therefore measures to employ more judicious use of these agents would be welcomed [86–88]. A more amenable relationship between bacteria–virus co-offenders *S. pneumoniae* and Rhinovirus, and *Moraxella* and Adenovirus, is evident in their statistically correlated abundance in recurrent acute OM in one cross-sectional study. With a 25% co-occurrence of most abundant viruses Rhinovirus and Bocavirus within its healthy controls, this may be a baseline virome not displaying obligate pathogenicity [89]. This opportunism spotlights a realm of known unknowns within aetiological factors capable of tipping this dormant microbiome into disease.

### NP profiling and health

NP microbiota in children under 1 year with bronchiolitis dominated by *Haemophilus* and with low levels of CCL5, a β-chemokine, has linear positive correlation with hospital stay when a *Moraxella*-dominated profile is used as a control, and that this trend was maintained when an AN swab was utilized. The AN is a not entirely comparable microbiological niche, where staphylococcal species represent 40 % total abundance compared to >5% in the NP [90–92]. Further profiling of the AN in a similar cohort showed the lowest proportion of patients developing severe bronchiolitis in the *Moraxella*-dominant group (14%) compared with *Staphylococcus*-dominated (47%). It is intriguing to speculate whether a similar trend could be found further back in the nasal cavity [93]? Bronchiolitis treatment escalation to mechanical ventilation was predicted specifically and sensitively by a select panel of 25 metabolites, which in turn mirrored relative abundance of *S. pneumoniae*, thus promoting the metabolome as a bacterial marker [94]. A multivariate analysis of associations of the NP microbiome within patients showed positive association with *S. pneumoniae* and *H. influenzae* and *M. catarrhalis*, siblings, daycare use, rhinoviruses and enteroviruses. There was a corresponding negative association with *S. aureus* carriage, recent antimicrobials, and the 7-valent pneumococcal vaccine [95]. A similar profiling of NP microbiota and patient characteristics established a ‘high’ degree of accuracy in predicting lower RTI and length of hospital stay from a 29-point score derived of the most indicative bacteria and viruses and patient factors. Thus antimicrobials are strongly indicated to prevent severe pneumonia, but with an underlying need for a stratified approach. It found relative scarcity of *Dolosigranulum* and *Corynebacterium* spp. and to a slightly lesser extent *Moraxella* spp. in those escalated to intensive care compared with controls [96]. Characterization of the NP microbiomes’ ability to prevent or accelerate viral respiratory infection is frustrated by the heterogeneity of study methods, lack of data from adult populations and poor taxonomic resolution [97]. Furthermore, the immune response against viruses is in turn modulated by gut microbiota, susceptible to the same deleterious effects from antimicrobials [98]. Nevertheless, in the light of the coronavirus pandemic of 2020, a theoretical underpinning for protective NP profiles could not be more welcome.

### The nasopharyngeal microbiome and antimicrobial resistance

Pressure on the NP microbiome by antibiotics could yield less protective profiles. Alpha diversity decreases linearly with antibiotic doses, whilst significantly increased relative abundance of *Haemophilus* is found in children aged 1–6 who had received antibiotics in the preceding 3 months compared to those who had not [99]. Thus, even before resistant pathogens are detected, disease susceptible states can persist following antibiotic use. *S. pneumoniae* resistant to Amoxicillin, Erythromycin and Co-Trimoxazole persist at stable rates throughout the first year of life within the South African population despite pneumoccocal vaccination at 6 weeks, and despite only 4% of HIV-exposed infants receiving the recommended Co-trimoxazole prophylaxis [100, 101]. Whole-genome sequencing (WGS) is more sensitive for detection of potential pathogens in patients with recent antimicrobial use compared to conventional culture [102]. The advent of WGS has shown great promise for decision-making around patient isolation, with the use of rapid WGS to classify skin and gut commensals in one hospital leading to a net saving of €200,000in blocked beds over 6 months despite high sequencing costs [103]. Such de-escalation of care would be well adapted to the high antimicrobial use seen in the upper respiratory tract.

### Sampling the NP

Accurate sampling of the NP tract remains a challenge to establishing a baseline NP microbiome. Despite the technique of nasopharyngeal swabbing and washes being standardized by the World Health Organization (WHO), considerable heterogeneity had been noted within clinical practice during the COVID-19 pandemic [104–107]. The nasopharynx is defined as a subcomponent of the upper throat or posterior nasal cavity. However during a recent systematic review of the scientific literature using risk-of-bias assessments and quality checklists, the anterior nares and posterior oropharyngeal wall have been found to be included within the term ‘nasopharynx’ [108, 109]. Accuracy is important: alpha diversity indices for brushings of the inferior turbinate was increased vs. washings of the whole nasal cavity; whether this was due to a richer sampled environment or removal of pathogens with a greater range of mucosal adhesion is difficult to assess [110]. Biogeography of the sphenoethmoidal recess and middle meatus displayed similar ecosystems likely related to their ciliated pseudostratified columnar epithelium, and dissimilar to the nonkeratinized, squamous epithelial inhabitants of the AN [111, 112]. Such studies rely on specialist equipment and expertise to accurately sample different sites. In some studies, nasal washings have yielded a higher colonization rate than swabbing of the posterior NP, but with implications of discomfort and suitability for an older paediatric/adolescent population [113–115]. Interestingly, once at the nasopharynx, completing the mandatory rotations did not have a major effect upon discomfiture [116]. Contamination of sampling by sites encountered *en passant* has to be a key consideration when moving beyond single pathogen carriage to quantifying microbial communities. Otolaryngological expertise has been relied on to specifically sample the nasopharynx in isolation [117, 118]. Innovation for this problem may include a ‘punch mechanism’ swab or a retractable guard for the swab head to sample the back of the nasal cavity only. When removed, the samples may themselves display further interactions: broth enrichment has been shown to favour overgrowth of phyla that would be disadvantaged *in vivo* at the expense of pathogens, and storage conditions affect microbial profiles as detected by 16S rRNA gene sequencing [119, 120]. The limitations of conventional culture persist from the laboratory to the clinic, with pathogen detection on sinus culture being unable to identify patients who would develop RSS-consistent radiological changes [121]. For pathogens enmeshed within biofilm, fluorescent *in situ* hybridization (FISH) had around twice the sensitivity of culture, invoking deeper sampling techniques for these landscapes [122]. Similar challenges exist with viruses: amplification of the specific fragment of the genome of common respiratory viruses via PCR has a sensitivity as low as 53 % with enzyme linked immunosorbent assay, and 71% by PCR [123, 124].

### Conclusion and future steps

The NP is an emerging as an arena in the fight against pneumonia and upper RTIs, and a reservoir evolving specific resistance patterns [125]. Encouraging developments have included the increase in diversity and stability of the NP microbiome since introduction of 7- and 13-valent pneumococcal vaccines, preceding a reduction in the incidence of OM [126]. Ingestion of probiotic yoghurt shows a significant decrease in the prevalence of Gram-positive pathobionts in humans, whilst probiotic application of *Corynebacterium* strains has been shown to replace *S. aureus* in humans and reduce viral load, lung changes and weight loss during RSV infection in mice [127–129]. Moving from marksmanship of culprit pathogens to shotgun sequencing of a patients’ entire microbiota at time of acute illness will guide increasingly accurate and judicious treatment of NP-derived infections. WGS is already being used to trace epidemiological links within outbreaks such as COVID-19 [130, 131]. For more common infections, ‘syndromic panels’ to detect carriage of a wide array of microbiota direct from samples have been available for the last decade [132]. Initial data on non-rapid WGS, defining infection as patients with exponentially prevalent overgrowth of key pathogens, suggests it can yield greater sensitivity, if not specificity, than conventional culture. Marriage of these technologies could inform front-of-house clinical decisions for more common infections in future. The generation of patient-specific microbial profiles may be able to distinguish health from disease, resistance patterns and specific dysbioses through emerging point-of-care whole-genome sequencing and clinical prediction tools [133, 134]. Such profiles available to clinicians may aid in decision making around ventilation, detection of disease in other body systems, and even transplantation of new microbiomes [135–137].
